# Supplementary material for: Squeezing microwaves by magnetostriction
Source: Natl Sci Rev. 2022 Nov 3;10(5):nwac247. doi: 10.1093/nsr/nwac247 (PMC10205500; doi:10.1093/nsr/nwac247)
Supplement: nwac247_Supplemental_File [file nwac247_supplemental_file.pdf]

Supplementary Material for
Squeezing Microwaves by Magnetostriction

Jie Li,[1,*] Yi-Pu Wang,[1] J. Q. You,[1] and Shi-Yao Zhu[1]

[1]*Interdisciplinary Center of Quantum Information, Zhejiang Province Key Laboratory of Quantum Technology and Device, and State Key Laboratory of Modern Optical Instrumentation, School of Physics, Zhejiang University, Hangzhou 310027, China*

EXPERIMENTAL SETUP FOR REALIZING THE PROTOCOL

Here we provide a specific experimental setup that is promising to realize our protocol. The setup and the description of the system are given in Fig. S1. It should be noted that, although a YIG sphere is adopted in this specific setup, our model and the corresponding results are generally valid for any magnon mode and mechanical mode that have a radiation pressure-like dispersive interaction, but not limited to a specific ferromagnet of a certain shape, as emphasized in the main text.

DERIVATION OF THE LINEARIZED QLES

From the Hamiltonian (1) in the main text, and by including the dissipation and input noise of each mode, we obtain the quantum Langevin equations (QLEs) in the frame rotating at the drive frequency ω_d

$$\begin{aligned} \dot{q} &= \omega_b p, \qquad \dot{p} = -\omega_b q - \gamma p - G_0 m^{\dagger} m + \xi, \\ \dot{m} &= -\Big(\frac{\kappa_m}{2} + i\Delta_m\Big) m - iga - iG_0 mq + \Omega + \sqrt{\kappa_m} m^{\rm in}, \\ \dot{a} &= -\Big(\frac{\kappa_a}{2} + i\Delta_a\Big) a - igm + \sqrt{\kappa_1} a_1^{\rm in} + \sqrt{\kappa_2} a_2^{\rm in}, \end{aligned} \tag{S1}$$

where γ is the mechanical damping rate, $\Delta_m = \omega_m - \omega_d$, and $\Delta_a = \omega_a - \omega_d$ are the detunings with respect to the magnon drive field. ξ denotes a Brownian stochastic force and describes a Gaussian quantum stochastic process, which is non-Markovian by nature, but it can be assumed Markovian for a large mechanical quality factor $Q \gg 1$ [1]. In this case, it becomes δ-correlated: $\langle \xi(t)\xi(t') + \xi(t')\xi(t)\rangle/2 \simeq \gamma[2\bar{n}_b(\omega_b) + 1]\delta(t{-}t')$. $m^{\rm in}$ and $a_j^{\rm in}$ $(j=1,2)$ are input noise operators for the magnon and cavity mode, respectively. $a_1^{\rm in}$ is the input noise entering the connector port of the microwave cavity, through which the output field is sent into a vector network analyser, or a homodyne detection scheme, and the corresponding external coupling rate is κ_1. $a_2^{\rm in}$ is the input noise describing all the other decay channels with a total decay rate $\kappa_2 \equiv \kappa_a - \kappa_1$. $m^{\rm in}$ and $a_j^{\rm in}$ are zero-mean and possess the following nonzero correlation functions [2]: $\langle m^{\rm in}(t)\, m^{\rm in\dagger}(t')\rangle = [\bar{n}_m(\omega_m) + 1]\,\delta(t{-}t')$, $\langle m^{\rm in\dagger}(t)\, m^{\rm in}(t')\rangle = \bar{n}_m(\omega_m)\,\delta(t{-}t')$, and $\langle a_j^{\rm in}(t)\, a_j^{\rm in\dagger}(t')\rangle = [\bar{n}_a(\omega_a) + 1]\,\delta(t{-}t')$, $\langle a_j^{\rm in\dagger}(t)\, a_j^{\rm in}(t')\rangle = \bar{n}_a(\omega_a)\,\delta(t{-}t')$, where $\bar{n}_j(\omega_j) = [\exp(\hbar\omega_j/(k_B T)) - 1]^{-1}$ $(j = b, m, a)$ are the equilibrium mean thermal phonon, magnon, photon number, respectively, with k_B the Boltzmann constant and T the environmental temperature.

To obtain sufficiently strong magnomechanical nonlinearity, we use a strong pump for the magnon mode. This results in a large amplitude of the magnon mode $|\langle m\rangle| \gg 1$, and further owing to the cavity-magnon state-swap interaction, the cavity field also gets a large amplitude. This validates the linearization treatment of the system dynamics, where we can write each mode operator as a fluctuation operator around its semiclassical average $O = \langle O\rangle + \delta O$, $(O = q, p, m, a)$, and neglect small second-order fluctuation terms. Substituting those linearized mode operators into Eq. (S1), we obtain two sets of equations for system averages and for quantum fluctuations. The solutions of the former allow us to define the system's working point, e.g., evaluate the effective detuning of the magnon mode and the effective magnomechanical coupling rate, while the solutions of the latter enable us to study the noise properties of each mode, which is the core of the

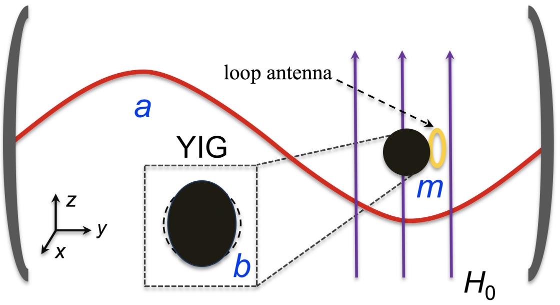

FIG. S1. Experimental setup of a cavity magnomechanical system. A YIG sphere is placed inside a microwave cavity near the maximum magnetic field of the cavity mode (in the x direction) and simultaneously in a uniform bias magnetic field (in the z direction). The magnon mode couples to the microwave cavity mode via the magnetic dipole interaction and to a vibrational phonon mode of the sphere by the nonlinear magnetostrictive interaction. The magnon mode is driven by a strong microwave field (with its magnetic field in the y direction) via a small loop antenna to enhance the magnomechanical coupling. The bias magnetic field, the drive magnetic field, and the magnetic field of the cavity mode are mutually perpendicular at the location of the YIG sphere. The cavity output microwave field is sent into a homodyne detection device for subsequent measurements.

* jieli007@zju.edu.cn

work. The QLEs for the quantum fluctuations are

$$\begin{aligned}
\delta\dot{q} &= \omega_b \delta p, \\
\delta\dot{p} &= -\omega_b \delta q - \gamma \delta p - G^* \delta m - G \delta m^\dagger + \xi, \\
\delta\dot{m} &= -\Big(\frac{\kappa_m}{2} + i\tilde{\Delta}_m\Big)\delta m - ig\delta a - iG\delta q + \sqrt{\kappa_m} m^{\rm in}, \\
\delta\dot{a} &= -\Big(\frac{\kappa_a}{2} + i\Delta_a\Big)\delta a - ig\delta m + \sqrt{\kappa_1} a_1^{\rm in} + \sqrt{\kappa_2} a_2^{\rm in},
\end{aligned} \tag{S2}$$

where $\tilde{\Delta}_m = \Delta_m + G_0\langle q\rangle$ is the effective detuning of the magnon mode, which includes the frequency shift induced by the magnetostrictive interaction, $\langle q\rangle = -\frac{G_0}{\omega_b}|\langle m\rangle|^2$, and $G = G_0\langle m\rangle$ is the effective magnomechanical coupling rate, which is complex as seen from the expression of $\langle m\rangle$, i.e.

$$\langle m\rangle = \frac{(\kappa_a/2 + i\Delta_a)\Omega}{(\kappa_m/2 + i\tilde{\Delta}_m)(\kappa_a/2 + i\Delta_a) + g^2}. \tag{S3}$$

DRIFT MATRIX USED FOR CHECKING THE STABILITY OF THE SYSTEM

We are interested in the squeezing of the cavity output field in the steady state. This means the system becomes stable and the system parameters must satisfy the stability condition. The parameter regime, within which the system is stable, can be derived by using the Routh-Hurwitz criterion [3]. However, the inequalities are quite involved for our hybrid tripartite system, and one can hardly extract useful information. We therefore study it numerically by checking the eigenvalues of the drift matrix. The Routh-Hurwitz criterion for stability is equivalent to all the eigenvalues (real parts) of the drift matrix being negative, such that the system parameters approach their steady-state values when time goes to infinity.

The drift matrix can be extracted from the QLEs (S2), which can be written in the matrix form, $\dot{u}(t) = Au(t) + n(t)$, where $u(t)$ is the vector of our system coordinates, $u(t) = (\delta q, \delta p, \delta m, \delta m^\dagger, \delta a, \delta a^\dagger)^T$, $n(t)$ is the vector of input noises, $n(t) = (0, \xi, \sqrt{\kappa_m} m^{\rm in}, \sqrt{\kappa_m} m^{\rm in\dagger}, \sqrt{\kappa_1} a_1^{\rm in} + \sqrt{\kappa_2} a_2^{\rm in}, \sqrt{\kappa_1} a_1^{\rm in\dagger} + \sqrt{\kappa_2} a_2^{\rm in\dagger})^T$, and A is the drift matrix, given by

$$A = \begin{pmatrix}
0 & \omega_b & 0 & 0 & 0 & 0 \\
-\omega_b & -\gamma & -G^* & -G & 0 & 0 \\
-iG & 0 & -(i\tilde{\Delta}_m + \frac{\kappa_m}{2}) & 0 & -ig & 0 \\
iG & 0 & 0 & -(-i\tilde{\Delta}_m + \frac{\kappa_m}{2}) & 0 & ig \\
0 & 0 & -ig & 0 & -(i\Delta_a + \frac{\kappa_a}{2}) & 0 \\
0 & 0 & 0 & ig & 0 & -(-i\Delta_a + \frac{\kappa_a}{2})
\end{pmatrix}. \tag{S4}$$

[1] Benguria R and Kac M. Quantum Langevin Equation. *Phys Rev Lett* 1981; **46**: 1.

[2] Gardiner CW and Zoller P. *Quantum Noise*. Springer, Berlin, Germany, 2000.

[3] Gradshteyn IS and Ryzhik IM. *Table of Integrals, Series and Products*. Academic, Orlando, 1980. p. 1119.
